# Essential oil composition and antimicrobial potential of aromatic plants grown in the mid-hill conditions of the Western Himalayas

**DOI:** 10.1038/s41598-023-31875-3

**Published:** 2023-03-25

**Authors:** Shalika Rathore, Srijana Mukhia, Rakshak Kumar, Rakesh Kumar

**Affiliations:** 1 Agrotechnology Division, CSIR-Institute of Himalayan Bioresource Technology, Post Box No. 6, Palampur, 176 061 Himachal Pradesh India; 2 Biotechnology Division, CSIR-Institute of Himalayan Bioresource Technology, Post Box No. 6, Palampur, 176 061 Himachal Pradesh India; 3 Academy of Scientific and Innovative Research (AcSIR), Ghaziabad, 201 002 India; 4 Guru Nanak Dev University, Amritsar, 143 005 Punjab India

**Keywords:** Biochemistry, Microbiology, Plant sciences

## Abstract

Essential oils are highly concentrated natural extracts obtained from plants, rich in bioactive constituents with antimicrobial properties, but the distinctive climate of the Western Himalayan region influences the same. Aromatic and medicinal plants, viz., *Origanum majorana, Origanum vulgare*, *Cymbopogon winterianus*, *Pelargonium graveolens*, and *Nepeta cataria* were grown in the foothills of the Western Himalayan condition and evaluated for essential oil content, composition, and their effect on some of the most common pathogenic microorganisms. The essential oil content (%) was 0.77, 0.45, 1.37, 0.15 and 0.17% in *O. majorana, O. vulgare*, *C. winterianus*, *P. graveolens*, and *N. cataria,* respectively. The major essential oil constituents of the isolated oils were terpinen-4-ol, thymol, citronellal, citronellol, and nepetalactone, contributing 41.24%, 31.81%, 43.13%, 43.35% and 91.43% in *O. majorana*, *O. vulgare*, *C. winterianus*, *P. graveolens*, and *N. cataria*, respectively. Well-diffusion assay revealed that the essential oil of *O. majorana* and *O. vulgare* was active against both the tested Gram-positive, viz., *Bacillus subtilis* MTCC 121, *Micrococcus luteus* MTCC 2470, and *Staphylococcus aureus* MTCC 96; and Gram-negative, viz., *Escherichia coli* MTCC 43, *Klebsiella pneumoniae* MTCC 109, and *Pseudomonas aeruginosa* MTCC 2453 bacteria, while the essential oil of *C. winterianus, P. graveolens,* and *N. cataria* showed activity against only some Gram-positive bacteria. Minimum inhibitory concentration (v/v) values indicated the highest efficacy of *O. majorana* essential oil against *B. subtilis* (0.5%), *M.* *luteus* (1%), and *S. aureus* (1%), while *O. vulgare* was most efficient to *E. coli* (2%) and *K. pneumoniae* (2%). *C. winterianus* essential oil did not inhibit any bacterial strains. *M. luteus* was susceptible to the essential oil of *P. graveolens* (1%) and *N. cataria* (0.5%) at low concentrations. Present findings showed the association between the chemical constituents’ profile of isolated essential oils from the Himalayan region and their antimicrobial activity, indicating their perspective to be utilized as antibacterial means.

## Introduction

The geographic location of the Indian Western Himalayan region is hot to sub-humid tropical in the southern tracks, warm-temperate to cool-temperate and cold alpine (2400–4800 m) mountain ranges in northern and eastern region. The region is rich in geological variability, biodiversity, and abundance of plant species. Usually, the geographical change and growing location of the plant affect the biosynthesis of chemical constituents and must be an important aspect in determining the content and chemical profile of essential oil^1^. The essential oils are fragrant fluids isolated from diverse portions of aromatic plants, potently producing distinctive plant taste or aroma. Essential oils play a well-known role in the food and perfume industry as flavoring and fragrance agents. More than 3000 essential oils are well-known, among which about 300 are of industrial connotation. Essential oils are a composite mixture of terpenes (monoterpenes and sesquiterpenes) and oxygenated derivatives^2^. The worth of a specific essential oil relies on the essential oil yield percentage, production rate, and demand. Essential oil yield varies in a broad range, i.e. 0.05–18.0% among various plant species^3^ and composition is a result of diverse compounds, even though in numerous plant species, only a single component may prevail over the others^4^. Aromatic plants have recently been reviewed for increased consideration by the research and scientific group because of their biological activities, viz., antimicrobial, and antioxidant properties, utilization in food, cosmetics, and perfumery industries^5^. In recent times, there has been a considerable increase in the market and utilization of many herbal plants, including *Origanum majorana* L. (marjoram), *Origanum vulgare* L. (oregano), *Cymbopogon winterianus* Jowitt ex Bor (Java citronella), *Pelargonium graveolens* L'Hér. (rose scented geranium), and *Nepeta cataria* L. (catnip). Since antiquity, *Origanum* species have been utilized in medicines and as spices primarily due to their essential oils, which contain a significant amount of two essential oil constituents, i.e. carvacrol and thymol. The other constituents in significant quantities include γ-terpinene, terpinene, cis-sabinene hydrate, and sabinene, which are being utilized in the food and medicine industry^6^. *O. majorana* has enormous industrial potential owing to its potent properties (antioxidant and antimicrobial) against various fungal and bacterial infections^7–9^. A few constituents, viz., cis-sabinene hydrate, terpinen-4-ol, and α-terpineol showed anti-inflammatory, antimicrobial, and anticancerous properties^10^. The *O.* *vulgare* plants possess thymol, p-cymene and γ-terpinene as major essential oil constituents^11^ while carvacrol, (*Z*)-α-bisabolene, caryophyllene oxide, linalyl acetate, and (E)-β*-*caryophyllene were recognized as the key components of *O. vulgare* essential oil^12^. Moreover, aromatic grass, i.e. java citronella is utilized for its essential oil which is rich in citronellal, citronellol, and geraniol being utilized in soap, incense, candles, perfumery, cosmetic, and flavouring industries; also has wider applications in pharmaceuticals^13^. Citronella essential oil chiefly contains monoterpenes with major contribution of aldehydes and alcohols^14,15^. The plant possesses a broad range of ethnopharmacological properties that rationalize its utilization in cosmetics, pest control, or as an anti-inflammatory agent^16^ and also possesses anti-inflammatory, antimicrobial, and antioxidant properties^17,18^. *P. graveolens* is an important scented aromatic plant that grows around the world for its essential oil, which is considered among the top 20 essential oils of the globe^19^. Furthermore, pelargonium essential oil is well-recognized for antimicrobial, anti-inflammatory, and anti-fungal properties^20,21^. The major constituents in *P. graveolens* essential oil are citronellol, geraniol, and 10-*epi*-*γ*-eudesmol^22^. *N. cataria*, a tropical aromatic plant, and its aerial parts (leaves and flowers) are utilized in the preparation of soup, sauce, and cheese. The medicinal usefulness of *Nepeta* species is generally ascribed towards the occurrence of flavonoids and volatile essential oils^23^. *N. cataria* essential oil is dominant with the presence of a constituent, i.e. 4a-α, 7-α, 7a-β nepetalactone (53.87%)^24^.

Several researchers have reported the compositional profile of essential oils from diverse regions around the globe. The composition varies depending on region, climatic conditions, grown variety, extraction/analytical methods, and vegetation period^25^. The utilization of man-made antioxidants, viz., butylated hydroxytoluene (BHT) and butylated hydroxyanisole (BHA), in the preservation of food has been reported to produce a few harmful effects^26^. Currently, the use of natural extracts has increased because of their potential to improve individual health and prevent pathologies, cancer, and atherosclerosis^8,27^. Several research studies have been carried out for essential oil composition determination in diverse aromatic and medicinal crops^28^.

The increasing interest in the replacement of man-made antimicrobial means with natural ones has fueled research activities on natural resources and essential oils, which have reported the antimicrobial activity to a varied extent^29–31^. Previous research has found that essential oils have antibacterial properties against animal/human pathogens, poisoning/spoilage of food, and plant bacterial pathogens^32,33^. Furthermore, the search for new antimicrobial agents has been necessitated because of the resistance developed to the presently available antimicrobial chemicals. There is a dearth of literature examining the production and antimicrobial potential of economically important aromatic crops grown under Western Himalayan conditions. Besides this, essential oil content and composition are also reported to be significantly affected by environmental conditions^34,35^, climate^36^, and growing conditions^37^. Therefore, the hypothesis of this study was that the essential oil content, profile, and bioactivity of economically important aromatic crops grown in the unique environmental conditions of the Western Himalayan region would be distinctive with varied constituent profiles and eventually lead to antimicrobial activity.

## Materials and methods

### Plant materials

Five plant species: *O. majorana O. vulgare*, *C. winterianus*, *P. graveolens*, and *N. cataria* were grown in the Western Himalayan region and experimental observation were recorded during 2020–2021 at an experimental farm of the Council of Scientific and Industrial Research-Institute of Himalayan Bioresource Technology, Palampur, India situated 1325 m above mean sea level (amsl) altitude (32°11′39"N latitude and 76°56′51"E longitude). The cultivated plants’ harvesting was done at the stage of their maturity. The experimental field study complies with relevant institutional guidelines and carried out in accordance with relevant regulations. The plant material identification was carried out by Dr Vikas Kumar (Plant Taxonomist) of the Environmental Technology Division of CSIR-IHBT. The specimens of *O. majorana* and *O. vulgare* have been deposited in the herbarium repository of CSIR-IHBT, Palampur, with voucher numbers 22070 and 22071, respectively. Variety Jor Lab C-5 of Java citronella (*C. winterianus*), and selections of Rose geranium (*P. graveolens*) selection IHBT/PG-1, catnip (*N. cataria*) selection IHBT/NC-1 were used in the present study and not deposited in herbarium repository of CSIR-IHBT as the plants were non native to Himalayan region and cultivated for commercial cultivation potential under homogeneous environmental conditions.

### Essential oil isolation and GC and GC/MS analysis

The plant parts (aerial) were harvested, and the material was brought for essential oil extraction to the laboratory. The essential oil extraction was initiated by hydrodistillation in a Clevenger-type apparatus by following a method similar to an earlier study^38^. GC and GC–MS analysis of essential oil of aromatic plants was initiated by a Shimadzu GC-2010 gas chromatograph (Shimadzu, Tokyo, Japan) which was connected with a flame ionization detector (FID) and a capillary column. The used column, carrier gas, flow rate, injector temperature, acquisition mass range, ionization energy, oven temperature conditions, and essential oil constituents’ quantification was done by a similar method as followed by^39^. Essential oil constituents’ identification was carried out by calculating retention indices (RI) by using a homologous hydrocarbons (C8–C24) series and peak area percentage in the chromatogram. The constituent’s identification was completed by comparing the RIs (calculated and literature) and mass fragmentation with the library database of NIST-MS (National Institute of Standards and Technology) Version 3.02^40,41^. The test samples were run in triplicates, and final observations were presented as mean ± standard deviation. The identified constituents were then analyzed in multivariate principal component analysis (PCA) software i.e. PAST3 (Paleontological Statistics Software Package for Education and Data Analysis version 3.22^42^ to evaluate the association of grown aromatic crops and essential oil constituents.

### Antibacterial assay

The essential oils of *O. vulgare*, *O. majorana, C. winterianus*, *P. graveolens*, and *N. cataria* were assessed for antibacterial activity against some bacterial opportunistic pathogens following the well-diffusion^43^ and broth microdilution methods^44,45^. The bacterial strains comprised of Gram-positive *Micrococcus* *luteus* MTCC 2470, *Bacillus subtilis* MTCC 121, and *Staphylococcus aureus* MTCC 96, and Gram-negative *Klebsiella pneumoniae* MTCC 109, *Escherichia coli* MTCC 43, and *Pseudomonas aeruginosa* MTCC 2453. The growing of broth temperature conditions, essential oil concentrations added into the holes punched, positive and negative control, essential oil diffusion and incubation duration and temperature were similar as used earlier^31^. The inhibition zones were recorded as per the earlier reported method^46^. The tests were run in triplicates, and final readings were presented as mean ± standard deviation. The minimum inhibitory concentration (MIC) values of the essential oil against the test strains were examined in a 96-well microtitre plate, where each essential oil was set in the concentration ranges of 8–0.125%. To the 50 µL of the essential oil, 50 µL of bacterial inoculum was added and kept at 37 °C for 12–24 h. This was followed by the addition of 30 µL resazurin (0.015%) and further incubation for 2–4 h. The MIC was considered the lowest concentration that showed no visible colour change from blue to pink.

## Results and discussion

### Climate and weather conditions

**Figure 1:**
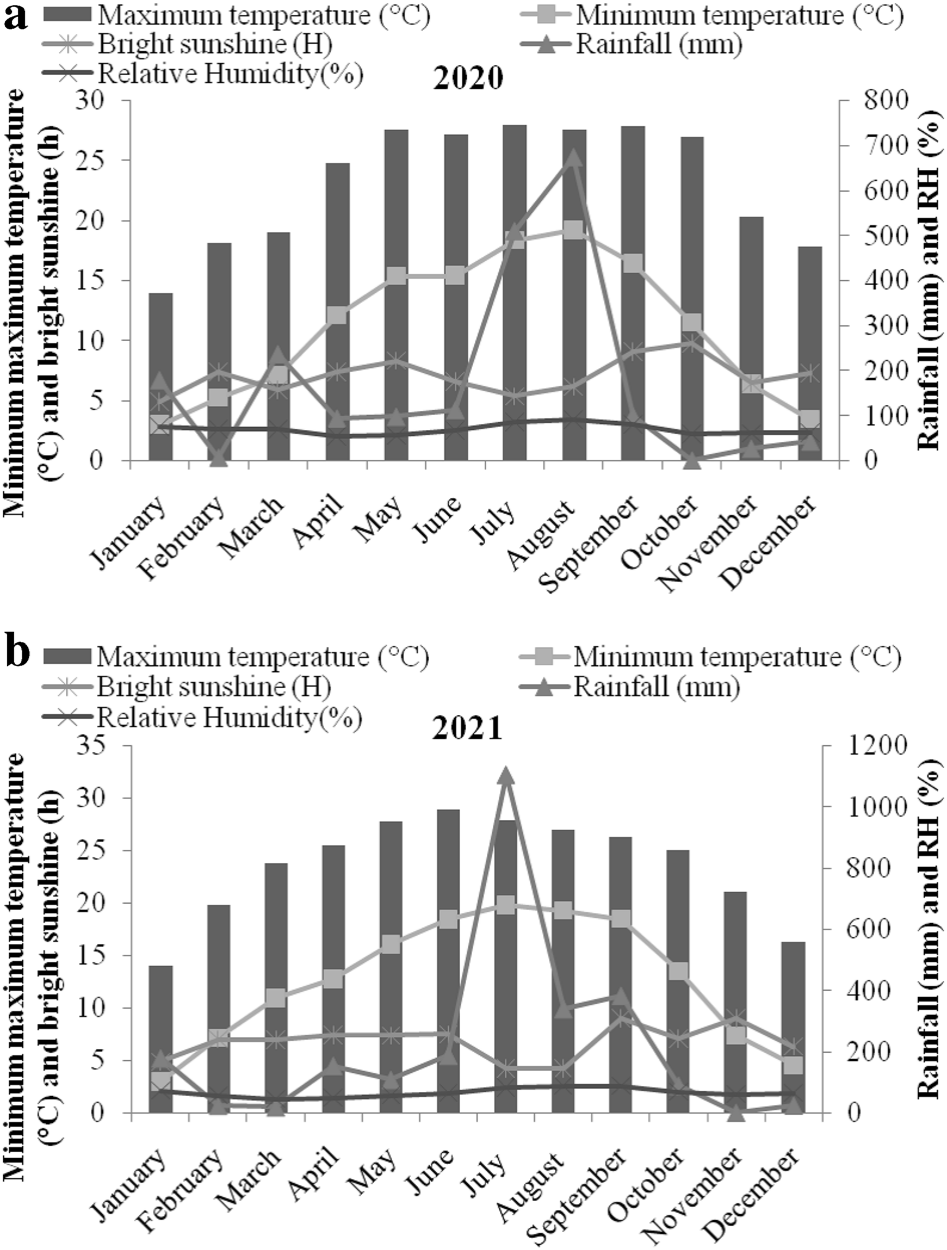
Mean weather conditions during the growth season of aromatic crops (**a**) 2020 and (**b**) 2021 at experimental site, Palampur, HP, India.

The climatic conditions of the experimental site were sub temperate sub humid, with a clay loam texture of the soil. Weather parameters, viz., temperature (°C) (minimum and maximum), relative humidity (RH%), and average bright sunshine (BSS) (hours) for two years of crop growth cycle were obtained from meteorological observatory of Chaudhary Sarwan Kumar Himachal Pradesh Agricultural University, Palampur, HP, through “Crop weather outlook”^47^ and are illustrated in Fig. 1. The mean maximum and minimum temperatures ranged from 3 to 28 and 3 to 29 °C during 2020 and 2021, respectively. Relative humidity was 54 to 90% and 50 to 89%, while BSS ranged from 5 to 10 and 4 to 9 h during 2020 and 2021, respectively. The maximum rainfall was recorded during August (673 mm) and July (1104 mm), while no rainfall was recorded during October and November in 2020 and 2021, respectively.

### Content and composition of essential oil

**Figure 2:**
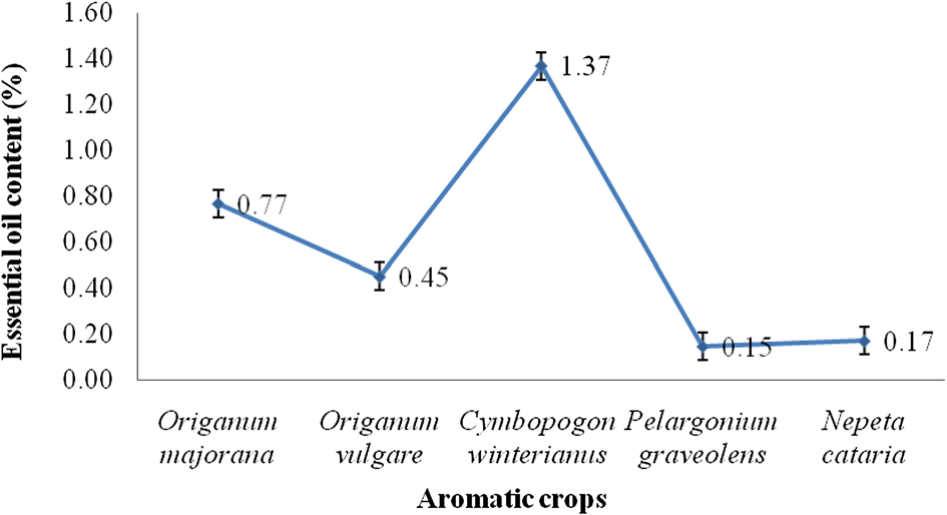
Essential oil content of aromatic medicinal plants grown in the mid hill conditions of the western Himalaya.

The essential oil content obtained from various aromatic plants is detailed in Fig. 2. The essential oil content of *O. majorana*, *O. vulgare, C. winterianus, P. graveolens,* and *N. cataria* was 0.77 ± 0.01, 0.45 ± 0.01, 1.37 ± 0.01, 0.15 ± 0.01, and 0.17 ± 0.01%, respectively. The major essential oil constituents obtained through hydro distillation are detailed in Table 1. The constituents contributing to more than 5% essential oil area in *O. majorana* were sabinene (5.4 ± 0.01%), β-myrecene (4.92 ± 0.19%), α-terpinene (4.02 ± 0.09%), α-phellandrene (4.13 ± 0.17%), cis-sabinene hydrate (10.26 ± 0.28%), terpinolene (5.02 ± 0.10%), 2-cyclohexen-1-ol (15.54 ± 0.46%), terpinen-4-ol (31.81 ± 0.34%), and caryophyllene (4.46 ± 0.04%). In marjoram essential oil, the key component (Table 1) was terpinen-4-ol (31.81 ± 0.34) followed by 2-cyclohexen-1-ol, cis-sabinene hydrate, sabinene, and terpinolene contributing for more than 5% of essential oil area while β-myrcene, α-terpinene, α-phellandrene, and caryophyllene contributed less than 5% in essential oil area. Terpinen-4-ol with cis-sabinene hydrate is accountable for the distinguishing fragrance and flavour; additionally, terpinene (α and γ) and terpinolene were other main components while carvacrol and thymol were present in lesser amounts^9^. Earlier study reported terpinen-4-ol as a major essential oil component while linalool, α-terpinene, α -terpinolene, α -terpineol, β-caryophyllene, α -terpinene, and spathulenol as minor constituents in marjoram^9^. Similarly, terpinen-4-ol followed by cis-sabinene hydrate was detailed as the foremost essential oil constituents of marjoram essential oil^48,49^. The essential oil constituents in *O. vulgare* were α-phellandrene (2.28 ± 0.02%), cis-sabinene hydrate (27.48 ± 0.17%), terpinen-4-ol (14.62 ± 0.12%), and thymol (43.13 ± 0.97%). The major essential oil of *O. vulgare* was thymol contributed to 43.13 ± 0.97% in the oregano essential oil followed by cis-sabinene hydrate and terpinen-4-ol. The present findings were similar to earlier report with thymol^49^ as the major essential oil component, while another recorded carvacrol as the major component^50^. The essential oil chemical profile of oregano is not homogeneous, as it includes two main chemotypes (thymol and carvacrol rich), while intermediate types contain both thymol and carvacrol. Oregano in the present study was rich in thymol and thus considered a thymol chemotype. The types with high content of p-cymene and γ-terpinene have also been identified in oregano of different origins^51^. Based on essential oil components, the oregano is divided in different categories: p-cymene > 14% and/or thymol > 6% is found only in Greek oregano, while borneol > 2% content was found only in Turkish oregano^52,53^.

**Table 1:** The main volatile essential oil compounds in aromatic medicinal plants grown in the mid hill conditions of the western Himalaya.

Essential oil compounds	RI		Aromatic medicinal plants					Identification methods
	Experimental	Literature	*Origanum majorana*	*Origanum vulgare*	*Cymbopogon winterianus*	*Pelargonium graveolens*	*Nepeta cataria*	
			Area (%)					
α-Thujene	925	924	0.76 ± 0.03	1.77 ± 0.01	–	–	–	RI, GC, GC/MS
α-Pinene	934	932	0.64 ± 0.01	0.83 ± 0.01	–	–	–	RI, GC, GC/MS
Sabinene	973	969	5.4 ± 0.01	–	–	–	–	RI, GC, GC/MS
β-Pinene	979	974	1.70 ± 0.01	–	–	–	–	RI, GC, GC/MS
β-Myrcene	986	988	4.92 ± 0.19	1.69 ± 0.01	–	–	–	RI, GC, GC/MS
α-Terpinene	1017	1014	4.02 ± 0.03	0.59 ± 0.01	–	–	–	RI, GC, GC/MS
Limonene	1030	1024	–		2.76 ± 0.01	–	–	RI, GC, GC/MS
α-Phellandrene	1026	1025	4.13 ± 0.17	2.28 ± 0.02	–	0.53 ± 0.11	–	RI, GC, GC/MS
cis-Sabinene hydrate	1030	1065	10.26 ± 0.28	27.48 ± 0.17	–	–	–	RI, GC, GC/MS
Terpinolene	1074	1086	5.02 ± 0.10	–	–	–	–	RI, GC, GC/MS
Linalool	1102	1095	–	–	0.72 ± 0.01	1.18 ± 0.01	–	RI, GC, GC/MS
trans-Sabinene hydrate	1086	1098	1.87 ± 0.76	–	–		–	RI, GC, GC/MS
Rose oxide B	1113	1106	–	–	–	2.54 ± 0.05	–	RI, GC, GC/MS
2-Cyclohexen-1-ol	1104	1118	15.54 ± 0.46	–	–	0.89 ± 0.11	–	RI, GC, GC/MS
Menthone	1132	1148	–	–	–	–	–	RI, GC, GC/MS
Citronellal	1155	1148	–	–	41.24 ± 0.37	–	–	RI, GC, GC/MS
Menthone < iso- >	1149	1158	–	–	–	–	–	RI, GC, GC/MS
Terpinen-4-ol	1185	1174	31.81 ± 0.34	14.62 ± 0.12	–	–	–	RI, GC, GC/MS
Menthan-2-one < cis-ρ- >	1178	1194	–	–	–	12.67 ± 0.06	–	RI, GC, GC/MS
Citronellol	1230	1223	–	–	9.91 ± 0.02	43.35 ± 0.48	–	RI, GC, GC/MS
Geraniol	1256	1249	–	–	16.8 ± 2.65	–	–	RI, GC, GC/MS
Phenol	1273	1264	–	–	0.51 ± 0.12	–	–	RI, GC, GC/MS
Citronellyl formate	1277	1271	–	–	–	20.99 ± 1.73	–	RI, GC, GC/MS
Citral < dimethoxy-(Z)- >	1305	1316	–	–	–	0.54 ± 0.21	–	RI, GC, GC/MS
Thymol	1296	1289	–	43.13 ± 0.97	–	–	–	RI, GC, GC/MS
Citronellyl acetate	1348	1350	–	–	2.90 ± 0.05	–	–	RI, GC, GC/MS
Neryl acetate	1378	1359	–	–	2.66 ± 0.57	–	–	RI, GC, GC/MS
Nepetalactone < 4aα,7α,7aβ- >	1380	1386	–	–		–	91.43 ± 0.30	RI, GC, GC/MS
β-Elemene	1388	1389	–	–	2.07 ± 0.40	–	–	RI, GC, GC/MS
Caryophyllene	1417	1408	4.46 ± 0.04	–	–	–	–	RI, GC, GC/MS
Citronellyl propanoate	1440	1444	–	–	–	2.26 ± 0.10	2.25 ± 0.02	RI, GC, GC/MS
Germacrene D	1482	1484	–	–	1.18 ± 0.03	–	–	RI, GC, GC/MS
β-Selinene	1498	1489	–	–	0.67 ± 0.45	–	–	RI, GC, GC/MS
γ-Cadinene	1519	1513	–	–	1.58 ± 0.02	–	–	RI, GC, GC/MS
Citronellyl butanoate	1526	1530	–	–		1.58 ± 0.10	–	RI, GC, GC/MS
Elemol	1554	1548	–	–	5.34 ± 0.20		–	RI, GC, GC/MS
Phenyl ethyl tiglate < 2- >	1597	1584	–	–	–	0.71 ± 0.48	–	RI, GC, GC/MS
Eudesmol < 10-epi-γ- >	1624	1622	–	–	–	2.71 ± 0.27	-	RI, GC, GC/MS
γ-Eudesmol	1642	1630	–	–	0.58 ± 0.04	–	–	RI, GC, GC/MS
α-Cadinol	1653	1652	–	–	1.37 ± 0.19	–	–	RI, GC, GC/MS
Eudesmol	1668	1661	–	–	4.66 ± 0.03	–	–	RI, GC, GC/MS
Citronellyl tiglate	1668	1666	–	–	–	1.33 ± 0.09	–	RI, GC, GC/MS

*RI* retention index, *GC* gas chromatography, *GC/MS* gas chromatography/mass spectrometry.

The values are mean ± standard deviation.

Similarly, the essential oil constituents identified in *C. winterianus* were limonene (2.76 ± 0.01%), citronellal (41.24 ± 0.37%), citronellol (9.91 ± 0.02%), geraniol (16.8 ± 2.65%), citronellyl acetate (2.90 ± 0.05%), neryl acetate (2.66 ± 0.57%), elemol (5.34 ± 0.20%), and eudesmol (4.66 ± 0.03%). The earlier studies depicted citronellal, citronellol, and geraniol as major constituents of *C. winterianus*^15^. However, the key constituent in *C. winterianus* essential oil is citronellal which is reported in current findings and gives a distinctive lemongrass aroma to the plant^15,54^. The composition of essential oil might be varied according to genotype and cultivars; lower percentage of citronellal and higher geraniol content in Medini cultivar of java citronella was recorded in earlier findings^13^. The major constituents identified in *P. graveolens* were rose oxide (2.54 ± 0.57%), menthan-2-one < cis-ρ- > (12.67 ± 0.06%), citronellol (43.35 ± 0.48%), citronellyl formate (20.99 ± 1.73%), citronellyl propanoate (2.26 ± 0.10%), and eudesmol < 10-epi-γ- > (2.71 ± 0.27%). The essential oil components of *P. graveolens* in the present study showed the highest amount of components such as citronellol (43.35 ± 0.48%) followed by menthan-2-one < cis-ρ- > (12.67 ± 0.06%), citronellyl formate (20.99 ± 1.73%), eudesmol < 10-epi-γ- > (2.71 ± 0.27%), rose oxide B (2.54 ± 0.05%), citronellyl propanoate (2.26 ± 0.10%), and citronellyl butanoate (1.58 ± 0.10%) while geraniol content was relatively low (< 0.50% not shown in data). Earlier studies also reported citronellol, geraniol, citronellyl formate, linalool, geranyl formate, menthone, isomenthone, and cis-rose oxide as major essential oil constituents^55^. However, *N. cataria* recorded nepetalactone < 4aα,7α,7aβ- > as a major essential oil constituent (91.43 ± 0.30%) followed by citronellyl propanoate (2.25 ± 0.02%) in Western Himalayan growing conditions. It can be noticed that few of the chief constituents were observed in more than one plant, viz., β-myrcene, α-terpinene, α-phellandrene, linalool, 2-cyclohexen-1-ol, terpinen-4-ol, citronellol, nepetalactone < 4aα,7α,7aβ- > , citronellyl propanoate, however, others were particular to specific plant species (Table 1). The essential oil components of *N. cataria* in the current finding showed the highest amount of nepetalactone < 4aα,7α,7aβ- > (91.43 ± 0.30%) followed by citronellyl propanoate (2.25 ± 0.02%); while the earlier findings reported 4a-α,7-α,7a-β-nepetalactone, 4a-α,7-β,7a-α-nepetalactone, and α-pinene as key essential oil constituents at 1810 m amsl altitude in sandy-loam slightly alkaline soil^56^.

The climatic and geographical variations produce significant discrepancy in the essential oil profile of aromatic plants grown under different soil and environmental conditions. In present study, terpinen-4-ol was recorded as major constituent of marjoram while in Mediterranean warm climatic condition of Turkey and temperate Argentinean conditions, thymol was the leading contributor to the essential oil^57,58^. The variations in the essential oil composition in the current study with Argentinean and Turkish condition might be ascribed to unique climatic situations and assorted agro-climatic stipulations of the growing region and acclimatized plants’ metabolism^8^. Similarly, the two different regions, viz., Nagarjun, Kathmandu (1537 m amsl) and Sanothimi, Bhaktapur (1336 m amsl) at different altitudinal variation in Nepal recorded slight variability in the content of terpinen-4-ol contributing 32.10 and 33.35% in the essential oil of marjoram, thus corroborating the present proposition that there might be a disparity in composition with geographical and atmospheric variability^59^.

Additionally, the geographical regions variability in Northern and Southern parts of Greece produced lower and higher thymol content, respectively, in essential oil of oregano^53^. However, carvacrol (70.0–77.4%) was the most dominant compound in neutral (pH 6.7) soils of Germany in a greenhouse setting followed by γ-terpinene and p-cymene^60^. Similarly, altitude is also an important environmental factor which plays a major role in manipulating the essential oil composition with high thymol at lower elevations and influencing the phenol pathway by thermal variability, thus increasing the total concentration of constituents with increased heat^61^. The different altitudinal ranges, viz., Auli (2744 m), Pithoragarh (1524 m), and Haldwani (412 m) in Western Himalayan region under natural field conditions specified thymol as the foremost component with higher concentration (52.83%) in intermediate altitude, i.e. 1524 m amsl and lowest in lower altitude^62^ thus corroborates the present findings with 43.13 ± 0.97% of thymol at an almost similar range of altitude at 1325 m amsl in the Western Himalayan region. The constituents 3'-terpinene and p-cymene (biosynthetic precursors) of carvacrol and thymol in oregano were influenced by environmental factors (thermal) thus influencing the qualitative composition of oregano^63^. Similarly, the genetic expression also influences the accumulation of key constituents; the dominant allele contributes to carvacrol, while the recessive one accumulates thymol^64^. Thus, in the present findings, environmental factors such as high bright sunshine hours (Fig. 1a,b) during the complete growth cycle may be responsible for the establishment of thymol-rich oregano. Carvacrol is not recorded in the present finding, which corroborates the earlier findings where thymol is produced at the expense of carvacrol and both varied inversely^61^. The different bioclimatic and geographical zones reported variation in constituents of oregano with a high concentration of carvacrol, linalyl acetate, (*Z*)-α-bisabolene, (*E*)-β*-*caryophyllene, and caryophyllene oxide in Iranian oregano^12^.

The growing conditions produced significant variation in essential oil composition; recorded citronellal, geraniol, and citronellol rich java citronella essential oil in Brazilian conditions^65^. However, java citronella recorded citronellal as major essential oil constituent followed by geraniol and citronellol^14^; the sequence of occurrence of constituents relative to their quantity is slightly different from current findings; the variability of essential oil might be because of diverse weather conditions in growing region. However, the key constituents were citronellal while citronellol and geraniol in *C. winterianus* under tropical monsoon climate with an average maximum temperature of 25 °C with hot and cold climate during summer and winter, respectively in Kumaon region, Uttarakhand under Indian Western Himalayan region^13^ which is comparable to present findings with similar range (30–45%) of major essential oil constituent i.e. citronellal which might be due to related environmental conditions viz., average temperature, sunshine, and rainfall settings (Fig. 1a,b). In contrast, the essential oil produced in much warmer conditions in Andhra Pradesh, India also recorded higher citronellal (50.93%) and comparatively lower citronellol and geraniol possibly be due differences in geographical location, climatic conditions, pedogenetic factors, season, and harvesting time^66^. Furthermore, the chemical constituents of the plant species differ depending on geographical origin, cultivars, cultivation method, photoperiod, harvest period and plant age^67^.

However, *P. graveolens* essential oil recorded in present study is at variance from that of Pauri Garhwal region of North India when citronellol, geraniol, linalool, citronellyl formate, and p-menthone were reported as major components while α-selinene and α-humulene as minor components^68^. Citronellol (51.0–63.4%) and isomenthone (9.8–17.8%) were reported as major constituents in cultivar “Kelkar”, while geraniol content (0.9% to 2.1%) was comparatively low^22^, which is corroborating the present findings with negligible geraniol content. The worth of rose scented geranium essential oil in the perfumery industry is primarily determined by C/G (citronellol/geraniol), which differed significantly according to cultivar, region, and location. The essential oil composition is affected by environmental conditions and reported citronellol and geraniol as key constituents of *P. graveolens* in North of Tunisia at 17 m amsl altitude^69^ while Egyptian *P. graveolens*, reported citronellol, trans-geraniol, and 10-epi-γ-eudesmol. In addition, the geraniol content is temperature dependent and generally decreases with falling night temperatures, whereas citronellol increases with a decrease in night temperatures^70–72^. In contrast, the cv. Kelkar recorded too high C/G ratio (30.19–70.44) in the essential oil; that too during summer, which is because of the presence of a lower geraniol area percentage as both the constituents are inversely related. Usually, rose scented geranium essential oil, which possesses an approximately equal amount of citronellol and geraniol (C/G between 0.5 and 2.0) is regarded as the greatest quality for commercial purposes^73^. The chemical profile of essential oil for geraniol content with previous findings^74,75^ showed sharp variability. The external factors, viz., varied developmental stages, harvest/collection times, soil and climatic conditions, cultivation region, geographic origin, or chemotype, may cause variability in essential oil (quantity and quality of constituents) of the identical species at diverse locations^76–78^. In sandy-loam neutral soils of Iranian conditions, the constituents such as α-pinene, β-pinene, and 4aα,7α,7aβ-nepetalactone were recorded at the full flowering stage of *N. cataria*^25^. The findings from Kashan, Iran, at 1550 m amsl, reported similar content of components of *N. cataria*^79^ as detected in the present study, which might be due to similar altitudinal and climatic conditions of the current study site. The earlier findings reported 4a-α,7-α,7a-β-nepetalactone, 1,8-cineole, and 4a-α, 7-β, 7a-α-nepetalactone as key constituents in the wild-growing *N. cataria* in Northern region of Iran^80^ while nepetalacones, β-caryophyllene and caryophyllene oxide were identified as chief constituents of the essential oil of *N. cataria* collected from Cordoba province of Argentina^81^. The quantitative and qualitative differences in essential oil composition may be because of chemotypes, mode of distillation, geographical and climatic factors^80^. The constituent profile of essential oil might be influenced by the phenological stages and geographical conditions of the growing region^32,82^, environmental conditions, plant nutritional status, season, and others^83^. Conversely, a few other studies reported deficiencies in nepetalactones but reported thymol^84^ and 1,8-cineole^85^ as the most abundant constituent of the *N. cataria* essential oil. The chief constituent reported was pinene (α and β) in the essential oil, which showed a gradual increase following the maturation of the plant^25^. Some of the key essential oil constituents were comparable to a few of the previously reported essential oils isolated from plants but differed from some of the other studies around the world. The geographical and growing location variation affected the biosynthesis of chemical constituents of *N. cataria*, reported 4aα,7α,7aβ-nepetalactone as a major constituent which has industrial utilization and chemotaxonomic markers in the essential oil.

The most important constituents of essential oils primarily belong to seven chemical groups: monoterpenes (α-thujene, sabinene, α-pinene, β-myrcene, β-pinene, α-terpinene, limonene, α-phellandrene, terpinolene), bicyclic monoterpenoids (cis & trans -sabinene hydrate), acyclic monoterpenoids (linalool), cyclic monoterpenoids (rose oxide b, 2-cyclohexen-1-ol, menthone, menthone, citronellal, isomenthone, menthan-2-one < cis-ρ- > , phenol/2-methoxy-3-(2-propenyl)-, citronellyl formate, citral < dimethoxy-(z)- > , thymol, citronellyl acetate, neryl acetate, nepetalactone < 4aα,7α,7aβ- >), alcoholic monoterpenoids (terpinen-4-ol, citronellol, geraniol), sesquiterpenes (β-elemene, caryophyllene, germacrene D, γ-cadinene, α-cadinol) and sesquiterpenoids (citronellyl propanoate, citronellyl butanoate, elemol, phenyl ethyl tiglate < 2- > , eudesmol < 10-epi-γ- > , γ-eudesmol, citronellyl tiglate) (Table 2). The chemical constituent profile of *O. majorana* and *O. vulgare* isolated essential oils remained in harmony with earlier reported studies^49,86^. The components of *C. winterianus, P. graveolens,* and *N. cataria* are similar to the earlier reported studies^15,25,51,53,68,87^.

**Table 2:** The grouped components (area %) of essential oil in aromatic medicinal plants grown in the mid hill conditions of the western Himalaya.

Grouped components	Aromatic crops				
	*O. majorana*	*O. vulgare*	*C. winterianus*	*P. graveolens*	*N. cataria*
Monoterpenes	26.59 ± 0.12	7.16 ± 0.04	2.76 ± 0.02	0.53 ± 0.03	‒
Bicyclic monoterpenoids	12.13 ± 0.05	27.48 ± 0.17	‒	‒	‒
Acyclic monoterpenoids	‒	‒	0.72 ± 0.03	1.18 ± 0.02	‒
Cyclic monoterpenoids	15.54 ± 0.06	43.13 ± 0.24	49.38 ± 0.36	37.63 ± 0.67	91.43 ± 0.45
Alcoholic monoterpenoids	31.81 ± 0.16	14.62 ± 0.21	26.71 ± 0.27	43.35 ± 0.80	‒
Sesquiterpene	4.46 ± 0.02	‒	3.43 ± 0.03	‒	‒
Sesquiterpenoids	‒	‒	11.95 ± 0.25	7.26 ± 0.04	2.25 ± 0.02
Total	90.53	92.39	94.95	89.95	93.68

The values are mean ± standard deviation.

### Antibacterial activity

**Figure 3:**
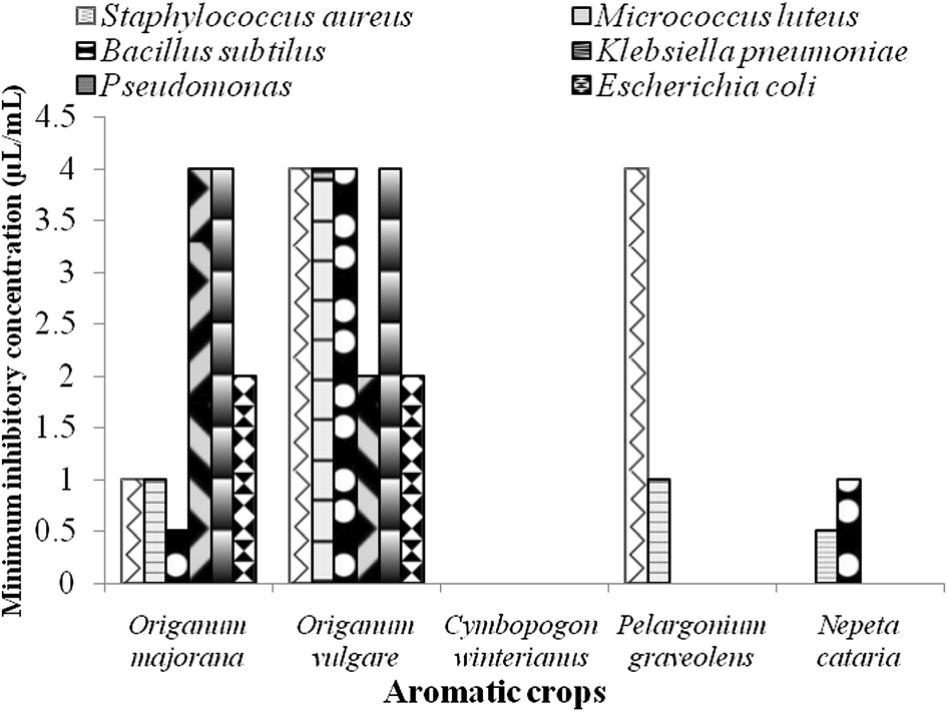
Minimum Inhibitory Concentration (MIC) (% v/v) values of essential oils against the bacterial strains. The experiment was repeated in triplicate on three separate occasions.

The well-diffusion assay revealed the strong antibacterial activities of *O. majorana* and *O. vulgare* against all the tested Gram-positive strains viz., *B. subtilis* MTCC 121 (6–14.33 mm), *M.* *luteus* MTCC 2470 (8.66–14.66 mm) and *S. aureus* MTCC 96 (7.5–8.66 mm), and the Gram-negative strains *E. coli* MTCC 43 (6–10 mm) and *K. pneumoniae* MTCC 109 (6–11 mm) (Table 3). These essential oils exhibited lesser activity against the Gram-negative *P. aeruginosa* MTCC 2453 (3.33–5.66 mm). However, only *M.* *luteus* and *S. aureus* fell under the sensitive category towards *O. majorana*, according to^46^. Earlier findings reported the efficacy of *O. majorana* essential oil in opposition to both the Gram-positive and Gram-negative bacteria, with good activities against *S. aureus* and *Bacillus* sp^59,88^. Likewise, the strains *M.* *luteus, B. subtilis, E. coli,* and *K. pneumoniae* may be marked to fall under the sensitive category towards *O. vulgare*. Similar to our findings, in an earlier report, the *O. vulgare* essential oil showed higher inhibitory activity against the Gram-positive bacteria than the Gram-negative ones^89^. On the other hand, *C. winterianus* and *N. cataria* showed lower inhibition zones against *B. subtilis* MTCC 121(2–4 mm) and *M.* *luteus* MTCC 2470 (3–3.33 mm), while no activities were observed against any other strains (Table 3). The essential oil of *P. graveolens* also exhibited very low activity against *M.* *luteus* MTCC 2470 (3 mm) and *S. aureus* MTCC 96 (4 mm) (Table 3). In case of *C. winterianus*, *P. graveolens* and *N. cataria*, the Gram-positive bacterial strains fell under the ‘not sensitive’ category. Also, these essential oils showed no antibacterial activities against the Gram-negative bacteria in the qualitative plate assay (Table 3). The results of well-diffusion assay demonstrate the essential oils of *O. majorana* and *O. vulgare* as potential antibacterial agents, among others. The MIC (% v/v) of each of these essential oils for antagonistic activity against the test bacterial strains was determined that show the lowest MIC values of *O. majorana* against the Gram-positive *B. subtilis* (0.5%), *M.* *luteus* (1%) and *S. aureus* (1%) (Fig. 3). According to the broth microdilution method, a lower MIC value corresponds to better activity of the test compound^90^. Among Gram-negative bacteria, *E. coli* was more sensitive (2% MIC) to *O. majorana* than *K. pneumoniae* (4%) and *P. aeruginosa* (4%) (Fig. 3)*.* In *O. vulgare*, the Gram-negative Strains *E. coli* and *K. pneumoniae* showed higher sensitivity (2% MIC) than *P. aeruginosa* (4% MIC) and all the tested Gram-positive strains (4% MIC) (Fig. 3). The results signify that the essential oils of *O. majorana* and *O. vulgare* showed inhibitory activities against both the Gram-positive and Gram-negative strains. *C. winterianus* did not show any inhibitory activity in the microdilution assay. Contrary to this, the essential oil of citronella has been reported with bactericidal activity against human pathogenic strains^91^. Interestingly, *M.* *luteus* was found to be more susceptible to *P. graveolens* (1%) and *N. cataria* (0.5%) in the microdilution assay. Earlier, *P. graveolens* essential oil came out to be ineffective against bacterial pathogens at lower concentrations^92^. *B. subtilis* also showed susceptibility to *N. cataria* (1%). *N. cataria* extracts have been reported to show high inhibitions of *B. subtilis* and *M. luteus* as indicated by the MIC values^93^. It might be inferred from the results that *P. graveolens* and *N. cataria* show potent inhibitory activity against some selective Gram-positive pathogenic strains.

**Table 3:** Antibacterial activity of the essential oils against Gram-positive and Gram-negative bacterial strains.

Essential oil	Gram-positive			Gram-negative		
	*Bacillus subtilis* MTCC 121	*Micrococcus luteus* MTCC2470	*Staphylococcus aureus* MTCC96	*Escherichia coli* MTCC 43	*Klebsiella pneumoniae* MTCC109	*Pseudomonas aeruginosa* MTCC 2453
*O. majorana*	6 ± 0	8.66 ± 0.5	8.66 ± 0.5	6 ± 0	6 ± 0	3.33 ± 0.5
*O. vulgare*	14.33 ± 0.5	14.66 ± 0.5	7.5 ± 0.5	10 ± 0	11 ± 0	5.66 ± 0.5
*C. winterianus*	4 ± 0	3.33 ± 0.5	–	–	–	–
*P. graveolens*	–	3 ± 0	4 ± 0	–	–	–
*N. cataria*	2 ± 0	3 ± 0	–	–	–	–
Streptomycin (1 mg/mL)	16.33 ± 0.5	6 ± 0	16.33 ± 0.5	16.33 ± 0.5	14 ± 0	7.66 ± 0.5

The values are mean ± standard deviation.

Values are means ± Standard Deviation (SD) of triplicate readings expressed in mm including 6 mm of well diameter.

### Principal component analysis (PCA)

**Figure 4:**
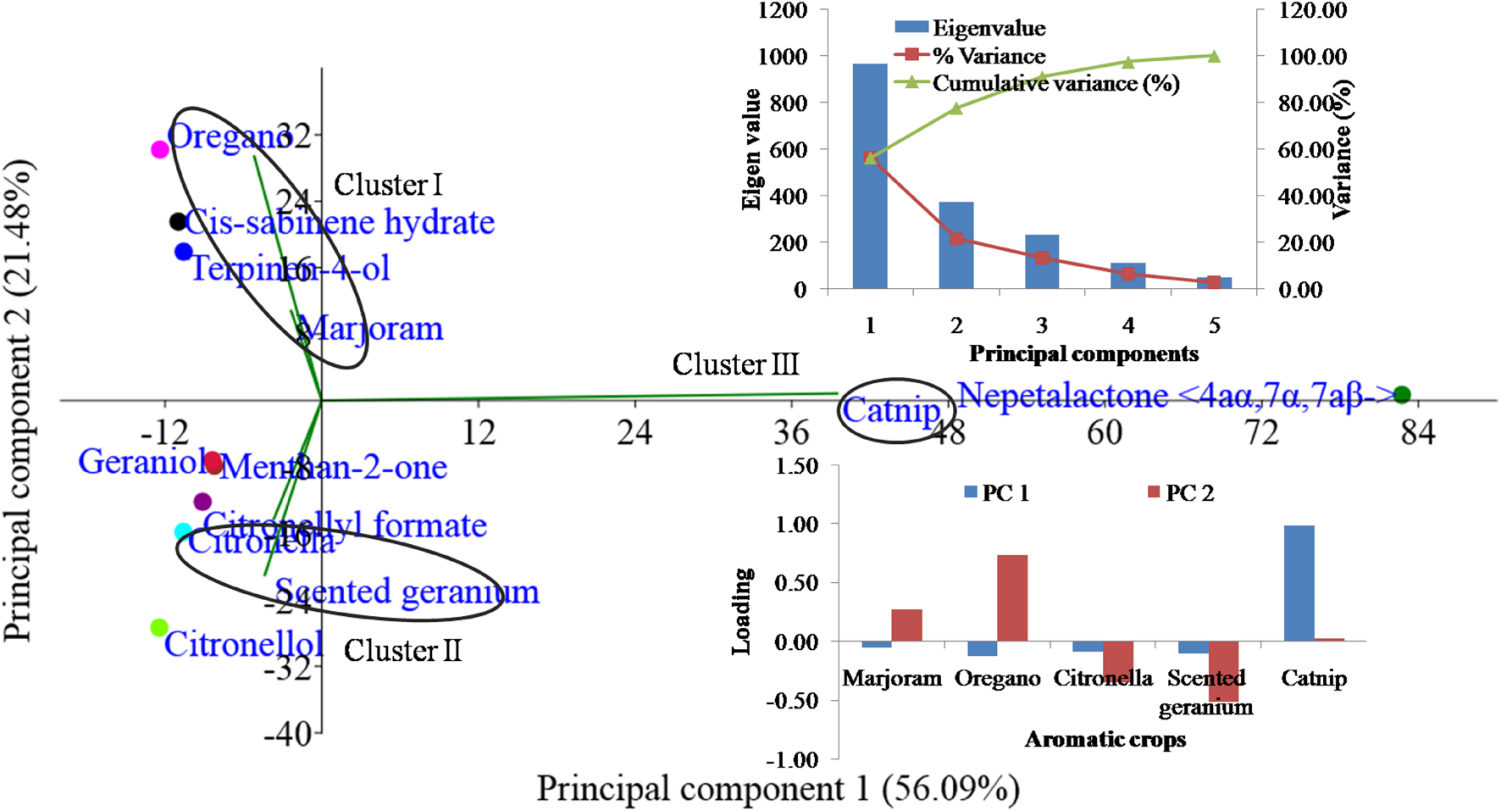
The multivariate analyses of mean value of major compounds of essential oil were conducted through principal component analysis. Principal component 1 and Principal component 2 jointly explained 77.57% of the total variation for aromatic plants grown in the western Himalaya; the eigenvalues and loading scores of the variables with PC1 and PC2 are presented at top right and bottom right corner of the figure, respectively.

Principal component analysis (PCA) was executed to assess the relation among aromatic crops and their essential oil constituents (Fig. 4). The PC analysis revealed 77.57% of variations elucidated by PC1 and PC2. Among all essential oil constituents, cis-sabinene hydrate, terpinen-4-ol, thymol, and nepetalactone < 4aα,7α,7aβ- > showed a positive association, while citronellal, menthan-2-one, citronellol, geraniol, and citronellyl formate showed negative association in PC2. However, in PC1 only nepetalactone < 4aα,7α,7aβ- > showed a positive association, while the rest of the constituents evidenced a negative association.

PC analysis separated oregano and marjoram crops, making them to be positioned on the same plane, while rose scented geranium and citronella were found to get positioned on the another plane. However, catnip was the only crop that appeared to be distinctive from other studied crops and lies alone in the separate plane (Fig. 4). Nepetalactone < 4aα,7α,7aβ- > was the only constituent that showed positive contribution and strong relationships with both PCs and a major essential oil constituent of catnip. The current study showed that the five PCs were highly illuminating and showed eigen values and thus contributed to about 97.29% of the whole variance of essential oil constituents. The experiential score plot of aromatic crops could be illustrated into three distinct clusters (Fig. 4 and Table 4). Cluster I include 10.26 and 27.48% of cis-sabinene hydrate, 31.81 and 14.62% of terpinen-4-ol in marjoram and oregano, respectively, while 43.13% of thymol only in oregano. Cluster I explained a higher concentration of terpinen-4-ol and thymol in marjoram and oregano, respectively. Cluster II includes crops viz., citronella and rose scented geranium which constitutes about 9.91 and 43.35% citronellol, while 41.24% and 16.80% citronellal and geraniol, respectively, and 20.99% of citronellyl formate only in rose scented geranium. Cluster II included distinctive constituents such as citronellal, menthan-2-one, citronellol, and citronellyl formate which were not observed in other clusters and thus grouped in cluster II. Furthermore, cluster III comprised 91.43% nepetalactone < 4aα,7α,7aβ- > which contributed to the majority of area percentage in essential oil and constituted an independent cluster of the single aromatic crop, i.e. catnip. The PCA separated treatments into three distinct clusters (Fig. 4), Cluster I exhibited cis-sabinene hydrate, terpinen-4-ol, thymol as major constituents, while Cluster II exhibited citronellal, menthan-2-one, citronellol, geraniol, and citronellyl formate. Cluster III was an independent cluster with the highest nepetalactone < 4aα,7α,7aβ- > content. The PC2 separated cis-sabinene hydrate, terpinen-4-ol, and thymol from other constituents and were placed within the positive end of PC2 with 21.54, 17.92, and 30.21 loadings, respectively. However, citronellal, menthan-2-one, citronellol, geraniol, and citronellyl formate were placed in the negative end of PC1 and PC2 with − 10.60 to − 15.81, − 8.23 to − 7.85, 12.45 to 27.33, − 8.38 to − 7.18, − 9.13 to − 12.19 and − 12.39 to 30.21 loading, respectively. However, nepetalactone < 4aα,7α,7aβ- > were placed in the positive end of both PCs with 82.74 and 0.69 loadings, respectively (Fig. 4). A noteworthy disparity in major essential oil profiles was recorded in different aromatic crops grown in the Western Indian Himalayan region, while some similarity was found in some minor constituents.

**Table 4:** Clusters variability in essential oil constituents (%) of the grown aromatic crops in the mid hills conditions of western Himalaya.

Essential oil constituents	Cluster I	Cluster II	Cluster III
Cis-sabinene hydrate	10.26‒27.48	0.00	0.00
Citronellal	0.00	0.00‒41.24	0.00
Terpinen-4-ol	14.62‒31.81	0.00	0.00
Menthan-2-one	0.00	0.00	0.00
Citronellol	0.00	9.91‒43.35	0.00
Geraniol	0.00	0.00‒16.80	0.00
Citronellyl formate	0.00	0.00	0.00
Thymol	0.00	0.00	0.00
Nepetalactone < 4aα,7α,7aβ- >	0.00	0.00	0.00‒91.43

## Conclusion

The present study suggests that isolated essential oils from five aromatic plants grown in the Western Himalayan region have essential oil quality according to the ISO standards and previously reported studies. Some of the extracted essential oils demonstrated remarkable antibacterial activities against selected opportunistic pathogenic strains. Consequently, these oils may perhaps become alternatives to synthesized antimicrobial compounds in controlling diseases causing agents. Though, more research on the security, safety, and toxicity of the studied essential oils ought to be carried out before further commercial usage (Supplementary Figure A).

## Supplementary Information

Supplementary Figures.

## Data Availability

The data analyzed during the current study available from the corresponding author on reasonable request.
