# Supplementary material for: Essential oil composition and antimicrobial potential of aromatic plants grown in the mid-hill conditions of the Western Himalayas
Source: Sci Rep. 2023 Mar 25;13:4878. doi: 10.1038/s41598-023-31875-3 (PMC10039882; doi:10.1038/s41598-023-31875-3)
Supplement: Supplementary file 1 — Supplementary Figures. [file 41598_2023_31875_MOESM1_ESM.doc]

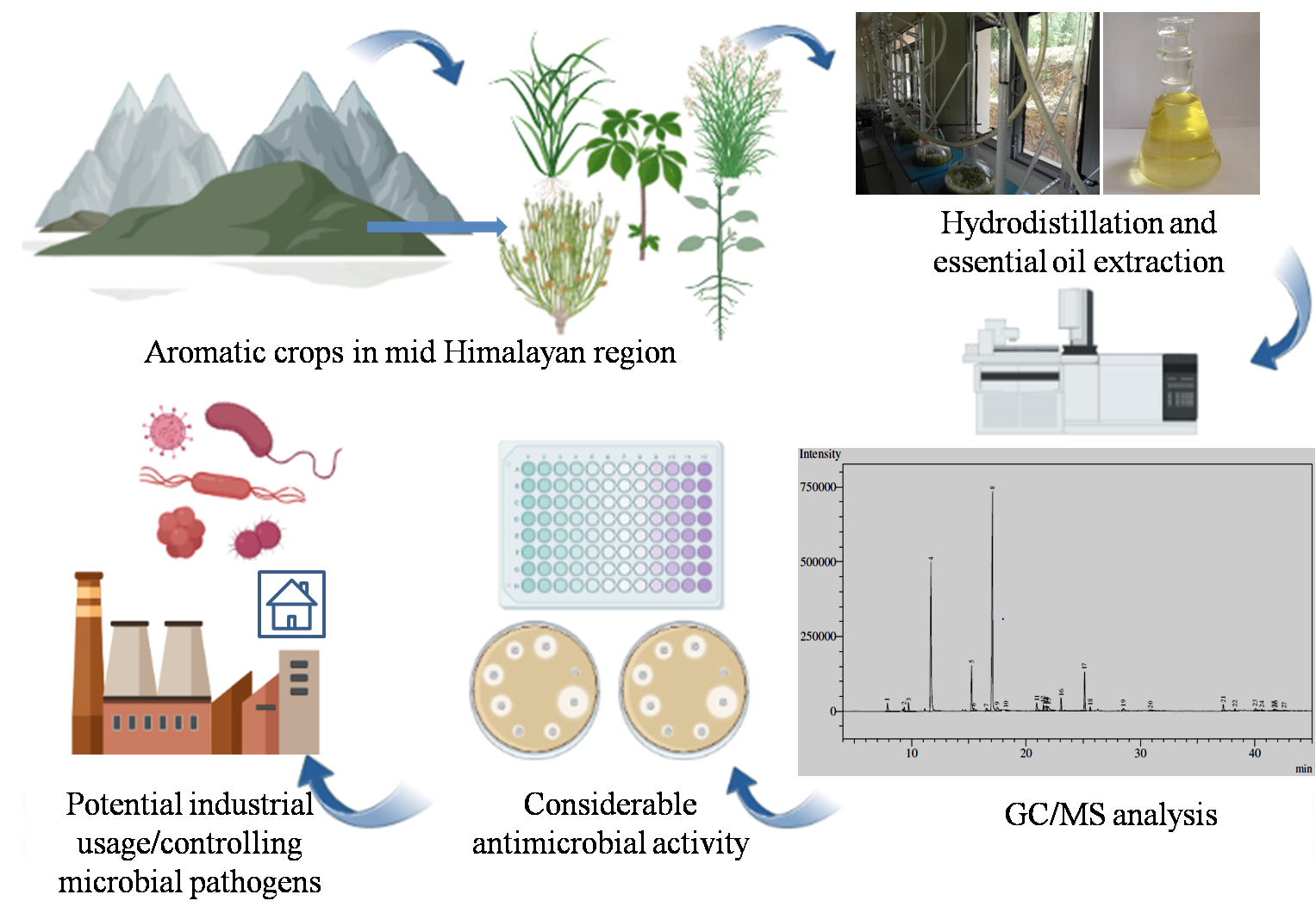


**Supplementary Figure A**. Representation of aromatic plants grown in Himalayan region for their essential oil quality, antimicrobial and industrial utilization potential. The graphic was created with BioRender.com
